# Supplementary material for: The Effect of Lumbopelvic Manipulation for Pain Reduction in Patellofemoral Pain Syndrome: A Systematic Review and Meta-Analysis of Randomized Controlled Trials
Source: Life (Basel). 2024 Jun 28;14(7):831. doi: 10.3390/life14070831 (PMC11278418; doi:10.3390/life14070831)

Supplemental Material

Table S1 PRISMA Checklist

Section and Topic	#	Checklist item	Location
**TITLE**			
Title	1	Identify the report as a systematic review.	Title
**ABSTRACT**			
Abstract	2	See the PRISMA 2020 for Abstracts checklist.	Abstract
**INTRODUCTION**			
Rationale	3	Describe the rationale for the review in the context of existing knowledge.	Introduction
Objectives	4	Provide an explicit statement of the objective(s) or question(s) the review addresses.	Introduction
**METHODS**			
Eligibility criteria	5	Specify the inclusion and exclusion criteria for the review and how studies were grouped for the syntheses.	Methods
Information sources	6	Specify all databases, registers, websites, organisations, reference lists and other sources searched or consulted to identify studies. Specify the date when each source was last searched or consulted.	Methods
Search strategy	7	Present the full search strategies for all databases, registers and websites, including any filters and limits used.	Methods, Table S2
Selection process	8	Specify the methods used to decide whether a study met the inclusion criteria of the review, including how many reviewers screened each record and each report retrieved, whether they worked independently, and if applicable, details of automation tools used in the process.	Methods
Data collection process	9	Specify the methods used to collect data from reports, including how many reviewers collected data from each report, whether they worked independently, any processes for obtaining or confirming data from study investigators, and if applicable, details of automation tools used in the process.	Methods
Data items	10a	List and define all outcomes for which data were sought. Specify whether all results that were compatible with each outcome domain in each study were sought (e.g., for all measures, time points, analyses), and if not, the methods used to decide which results to collect.	Methods

	10b	List and define all other variables for which data were sought (e.g., participant and intervention characteristics, funding sources). Describe any assumptions made about any missing or unclear information.	Methods Table 1
Study risk of bias assessment	11	Specify the methods used to assess risk of bias in the included studies, including details of the tool(s) used, how many reviewers assessed each study and whether they worked independently, and if applicable, details of automation tools used in the process.	Methods
Effect measures	12	Specify for each outcome the effect measure(s) (e.g., risk ratio, mean difference) used in the synthesis or presentation of results.	Methods
Synthesis methods	13a	Describe the processes used to decide which studies were eligible for each synthesis (e.g., tabulating the study intervention characteristics and comparing against the planned groups for each synthesis (item #5)).	Methods, Figure 1, Table 1, Table S3
	13b	Describe any methods required to prepare the data for presentation or synthesis, such as handling of missing summary statistics, or data conversions.	Methods
	13c	Describe any methods used to tabulate or visually display results of individual studies and syntheses.	Methods
	13d	Describe any methods used to synthesize results and provide a rationale for the choice(s). If meta-analysis was performed, describe the model(s), method(s) to identify the presence and extent of statistical heterogeneity, and software package(s) used.	Methods
	13e	Describe any methods used to explore possible causes of heterogeneity among study results (e.g., subgroup analysis, meta-regression).	Methods
	13f	Describe any sensitivity analyses conducted to assess robustness of the synthesized results.	Methods
Reporting bias assessment	14	Describe any methods used to assess risk of bias due to missing results in a synthesis (arising from reporting biases).	Methods, Figure S1, Table 3
Certainty assessment	15	Describe any methods used to assess certainty (or confidence) in the body of evidence for an outcome.	Methods
**RESULTS**			
Study selection	16a	Describe the results of the search and selection process, from the number of records identified in the search to the number of studies included in the review, ideally using a flow diagram.	Results, Figure 1, Table S2-3

	16b	Cite studies that might appear to meet the inclusion criteria, but which were excluded, and explain why they were excluded.	Results, Table S3
Study characteristics	17	Cite each included study and present its characteristics.	Results, Table 1
Risk of bias	18	Present assessments of risk of bias for each included study.	Figure S1, Table 2
Results of individual studies	19	For all outcomes, present, for each study: (a) summary statistics for each group (where appropriate) and (b) an effect estimates and its precision (e.g., confidence/credible interval), ideally using structured tables or plots.	Figure 2-5, Figure S2-3
Results of syntheses	20a	For each synthesis, briefly summarise the characteristics and risk of bias among contributing studies.	Results, Table 3
	20b	Present results of all statistical syntheses conducted. If meta-analysis was done, present for each the summary estimate and its precision (e.g., confidence/credible interval) and measures of statistical heterogeneity. If comparing groups, describe the direction of the effect.	Results, Figure 3-5, Figure S2-3
	20c	Present results of all investigations of possible causes of heterogeneity among study results.	Results, Figure 3-5, Figure S2-3
	20d	Present results of all sensitivity analyses conducted to assess the robustness of the synthesized results.	Results, Figure 3
Reporting biases	21	Present assessments of risk of bias due to missing results (arising from reporting biases) for each synthesis assessed.	Figure S1, Table 3
Certainty of evidence	22	Present assessments of certainty (or confidence) in the body of evidence for each outcome assessed.	Figure 3-5, Figure S2-3
**DISCUSSION**			
Discussion	23a	Provide a general interpretation of the results in the context of other evidence.	Discussion
	23b	Discuss any limitations of the evidence included in the review.	Discussion
	23c	Discuss any limitations of the review processes used.	Discussion
	23d	Discuss implications of the results for practice, policy, and future research.	Discussion

OTHER INFORMATION			
Registration and protocol	24a	Provide registration information for the review, including register name and registration number, or state that the review was not registered.	Methods
	24b	Indicate where the review protocol can be accessed, or state that a protocol was not prepared.	Methods, Table S2-3
	24c	Describe and explain any amendments to information provided at registration or in the protocol.	Methods, Table S2-3
Support	25	Describe sources of financial or non-financial support for the review, and the role of the funders or sponsors in the review.	Funding
Competing interests	26	Declare any competing interests of review authors.	Conflicts of interest
Availability of data, code and other materials	27	Report which of the following are publicly available and where they can be found: template data collection forms; data extracted from included studies; data used for all analyses; analytic code; any other materials used in the review.	Results, Table S2-3

Table S2 Keywords and search results in different databases

Database	Keyword	Filter	Date	Results
PubMed	(“lumbopelvic manipulation” OR “lumbopelvic thrust manipulation” OR “lumbosacral manipulation” OR “lumbar manipulation” OR “pelvic manipulation”) AND (“patellofemoral pain syndrome” OR “patellofemoral syndrome”)	NA	December 1, 2023	1943
Cochrane library	(“lumbopelvic manipulation” OR “lumbopelvic thrust manipulation” OR “lumbosacral manipulation” OR “lumbar manipulation” OR “pelvic manipulation”) AND (“patellofemoral pain syndrome” OR “patellofemoral syndrome”)	Title Abstract Keyword	December 1, 2023	763
Clinical trials.gov	(“lumbopelvic manipulation” OR “lumbopelvic thrust manipulation” OR “lumbosacral manipulation” OR “lumbar manipulation” OR “pelvic manipulation”) AND (“patellofemoral pain syndrome” OR “patellofemoral syndrome”)	Condition or disease	December 1, 2023	4
PEDro	(“lumbopelvic manipulation” OR “lumbopelvic thrust manipulation” OR “lumbosacral manipulation” OR “lumbar manipulation” OR “pelvic manipulation”) AND (“patellofemoral pain syndrome” OR “patellofemoral syndrome”)	Condition or disease	December 1, 2023	7

Table S3 Excluded studies and reasons

Citations	Reasons
Crowell MS, Wofford NH. Lumbopelvic manipulation in patients with patellofemoral pain syndrome. J Man Manip Ther. 2012 Aug;20(3):113-20. doi: 10.1179/2042618612Y.0000000002	Not a randomized controlled trial
Espí-López, G. V., Serra-Añó, P., Vicent-Ferrando, J., Sánchez-Moreno-Giner, M., Arias-Buría, J. L., Cleland, J., & Fernández-de-Las-Peñas, C. (2017). Effectiveness of Inclusion of Dry Needling in a Multimodal Therapy Program for Patellofemoral Pain: A Randomized Parallel-Group Trial. The Journal of orthopaedic and sports physical therapy, 47(6), 392–401. doi: 10.2519/jospt.2017.7389	Both experimental and control groups employed lumbopelvic manipulation
Shakouri, A., Kamali, F., Mohamadi, M., & Nouhi, E. (2023). Lumbopelvic manipulation alone versus combined with dry needling in physically active patients with patellofemoral pain syndrome: A randomized clinical trial. Journal of Bodywork and Movement Therapies.	Both experimental and control groups employed lumbopelvic manipulation
Hillermann, B. (2003). The effect of three manipulative treatment protocols on quadriceps muscle strength in patients with Patellofemoral Pain Syndrome (Doctoral dissertation).	Not a randomized controlled trial
Iverson CA, Sutlive TG, Crowell MS, Morrell RL, Perkins MW, Garber MB, Moore JH, Wainner RS. Lumbopelvic manipulation for the treatment of patients with patellofemoral pain syndrome: development of a clinical prediction rule. J Orthop Sports Phys Ther. 2008 Jun;38(6):297-309; discussion 309-12. doi: 10.2519/jospt.2008.2669	Not a randomized controlled trial
Kumar, R. M., & Shereief, B. (2022). Effect of Lumbopelvic Manipulation Versus Tibiofemoral Mobilization on Pain and Quality of Life in Patellofemoral Pain Syndrome. Indian Journal of Physiotherapy & Occupational Therapy Print-(ISSN 0973-5666) and Electronic–(ISSN 0973-5674), 16(2), 197-207.	Not a randomized controlled trial
Suter E, McMorland G, Herzog W, Bray R. Decrease in quadriceps inhibition after sacroiliac joint manipulation in patients with anterior knee pain. J Manipulative Physiol Ther. 1999 Mar-Apr;22(3):149-53. doi: 10.1016/S0161-4754(99)70128-4.	Not on patellofemoral pain syndrome
Grindstaff, T. L., Hertel, J., Beazell, J. R., Magrum, E. M., Kerrigan, D. C., Fan, X., & Ingersoll, C. D. (2012). Lumbopelvic joint manipulation and quadriceps activation of people with patellofemoral pain syndrome. Journal of athletic training, 47(1), 24-31. doi: 10.4085/1062-6050-47.1.24	Lack of reporting pain intensity
Hillermann, B., Gomes, A. N., Korporaal, C., & Jackson, D. (2006). A pilot study comparing the effects of spinal manipulative	Lack of reporting pain intensity

therapy with those of extra-spinal manipulative therapy on quadriceps muscle strength. Journal of manipulative and physiological therapeutics, 29(2), 145-149. doi: 10.1016/j.jmpt.2005.12.003

Figure S1. Summary of quality assessment of studies included in the meta-analysis using Cochrane risk of bias 2 tool

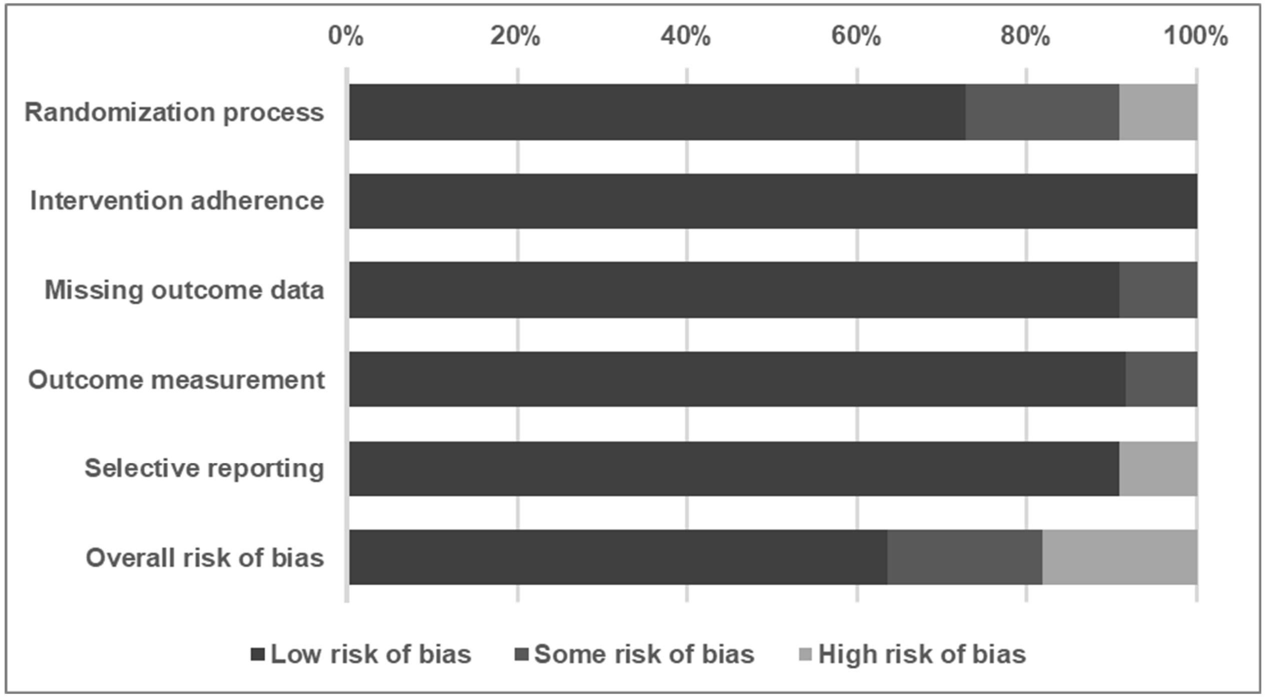

Figure S2. The results of sensitivity analysis using the one–study removal method

Study name	Statistics with study removed			
	Point	Lower limit	Upper limit	p-Value
Brantingham 2009	-0.766	-1.300	-0.231	0.005
Miller 2013	-0.734	-1.264	-0.203	0.007
Selvam 2013	-0.620	-1.125	-0.115	0.016
Al Abbad 2014	-0.761	-1.293	-0.229	0.005
Motealleh 2016	-0.601	-1.092	-0.110	0.016
Behrangrad 2017	-0.578	-1.050	-0.107	0.016
Azizi 2020	-0.772	-1.303	-0.240	0.004
Motealleh 2020	-0.681	-1.227	-0.134	0.015
Scafoglieri 2021	-0.671	-1.212	-0.130	0.015
D'Agati 2022	-0.849	-1.280	-0.418	0.000
Zafarian 2023	-0.736	-1.277	-0.196	0.008
	-0.706	-1.197	-0.214	0.005

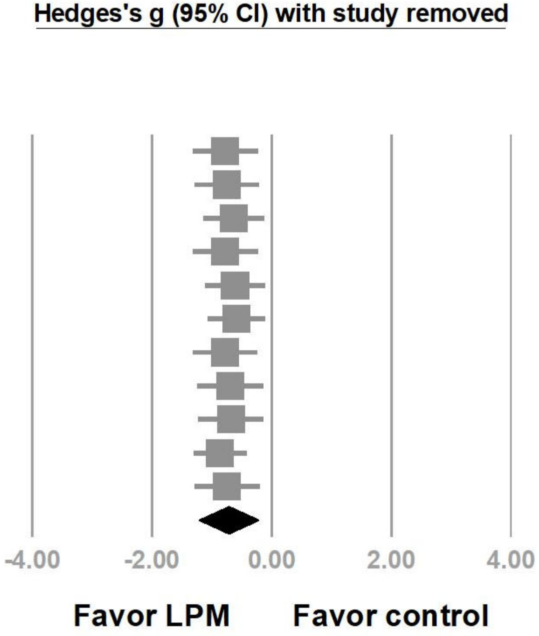

Figure S3. The subgroup analysis based on assessment time point

Group by<br>Assessment time point	Study name	Statistics for each study<br>Hedges's g	Lower limit	Upper limit	p-Value
Delay effect	Brantingham 2009	-0.141	-0.837	0.554	0.690
Delay effect	Behrangrad 2017	-2.047	-2.915	-1.179	0.000
Delay effect	Azizi 2020	-0.081	-0.778	0.616	0.820
Delay effect	Scafoglieri 2021	-1.052	-1.686	-0.417	0.001
Delay effect	Zafarian 2023	-0.420	-1.173	0.333	0.274
Delay effect		-0.723	-1.379	-0.067	0.031
Immediate effect	Miller 2013	-0.410	-1.352	0.533	0.394
Immediate effect	Selvam 2013	-1.586	-2.389	-0.782	0.000
Immediate effect	Al Abbad 2014	-0.160	-0.934	0.614	0.686
Immediate effect	Motealleh 2016	-1.812	-2.674	-0.951	0.000
Immediate effect	Motealleh 2020	-0.960	-1.574	-0.346	0.002
Immediate effect	D'Agati 2022	0.659	0.049	1.269	0.034
Immediate effect		-0.694	-1.474	0.086	0.081

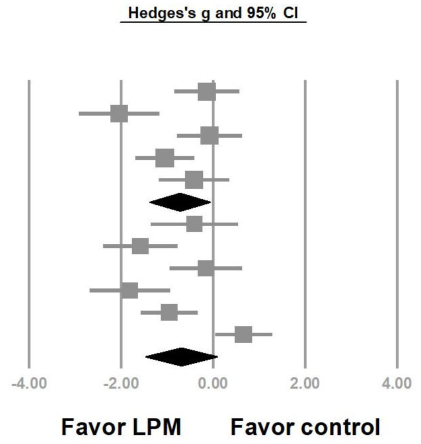

Figure S4. The funnel plot of the included trials

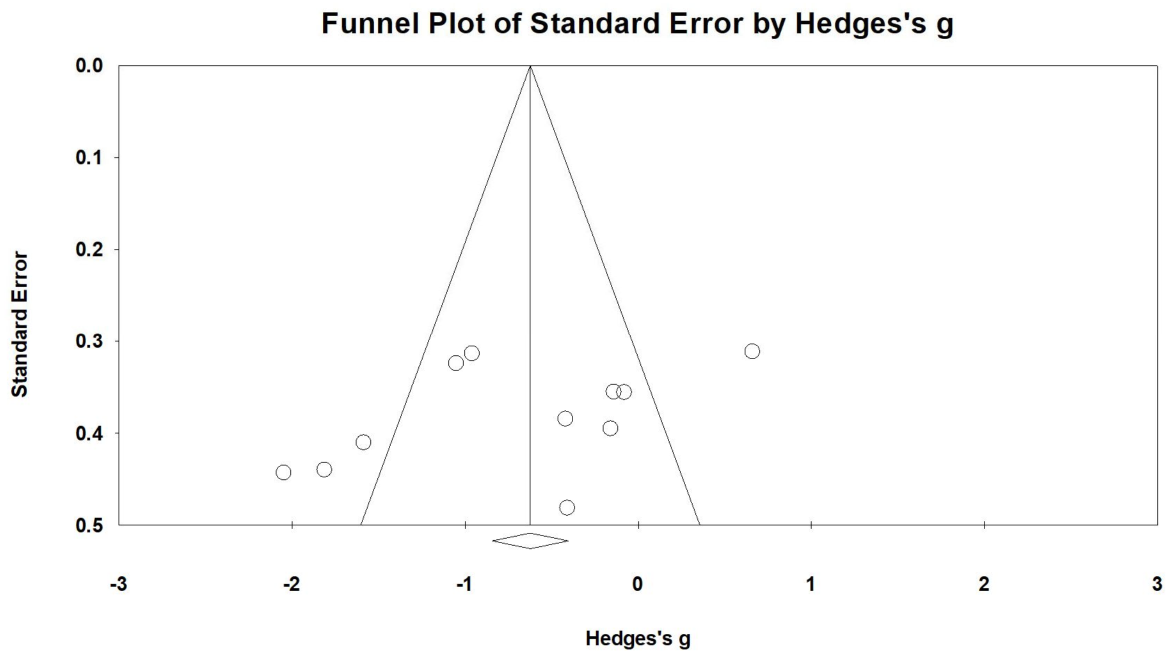

Supplement: Supplementary file 1 [file life-14-00831-s001.zip › life-3050463-supplementary.pdf]
